# Enhancing apoptotic cell clearance mitigates bacterial translocation and promotes tissue repair after gut ischemia-reperfusion injury

**DOI:** 10.3892/ijmm.2012.1044

**Published:** 2012-06-26

**Authors:** RONGQIAN WU, WEIFENG DONG, ZHIMIN WANG, ASHA JACOB, TIANPEN CUI, PING WANG

**Affiliations:** Laboratory of Surgical Research, The Feinstein Institute for Medical Research and Department of Surgery, Hofstra North Shore-Long Island Jewish School of Medicine, Manhasset, NY, USA

**Keywords:** milk fat globule-epidermal growth factor-factor 8, gut ischemia/reperfusion, apoptosis, bacterial translocation, vascular endothelial growth factor

## Abstract

A key aspect of intestinal ischemia/reperfusion (I/R) injury is the increased occurrence of apoptotic cell death in the gut. Insufficient clearance of apoptotic cells leads to increased inflammation and impaired tissue repair. Our recent studies have shown that administration of milk fat globule-epidermal growth factor-factor 8 (MFG-E8), a crucial molecule for apoptotic cell clearance, reduces apoptosis and inflammation under various disease conditions. The purpose of this study was to determine whether MFG-E8 reduces bacterial translocation and promotes tissue repair in a mouse model of gut I/R. Gut ischemia was induced by placing a microvascular clip across the superior mesenteric artery for 90 min in male adult mice. After removing the clip, recombinant murine MFG-E8 (rmMFG-E8) (0.4 μg/20 g BW) or normal saline (Vehicle) was intraperitoneally injected. At 4 h after reperfusion, apoptosis in the gut was measured by TUNEL staining. The mesenteric lymph node (MLN) complex was homogenized and plated on chocolate agar plates for bacterial culture. Neutrophil infiltration was assessed by examining myeloperoxidase (MPO) activity in the gut. Vascular endothelial growth factor (VEGF) levels in the gut, an indicator of tissue repair, were measured by western blotting. Out results showed that TUNEL-positive staining in the gut increased significantly in gut I/R vehicle-treated mice. Treatment with rmMFG-E8 markedly suppressed the number of apoptotic cells. Bacterial translocation to the MLN was minimal in sham mice, but was extensive in gut I/R vehicle-treated mice. rmMFG-E8 treatment significantly reduced bacterial translocation to the MLN. Similarly, gut I/R induced a significant increase in intestinal MPO activities in vehicle-treated mice. rmMFG-E8 treatment markedly reduced the increase in intestinal MPO activities after gut I/R. Intestinal levels of VEGF decreased significantly at 4 h after gut I/R. rmMFG-E8 treatment significantly increased intestinal VEGF levels. Thus, enhancing apoptotic cell clearance by rmMFG-E8 mitigates bacterial translocation, inhibits neutrophil infiltration and promotes tissue repair after gut I/R. Enhancing apoptotic cell clearance can be a novel concept in the treatment of gut I/R injury.

## Introduction

Intestinal ischemia is a common clinical problem occurring in many clinical settings such as superior mesenteric artery occlusion, hemorrhagic shock, cardiac insufficiency with associated low flow state, necrotizing enterocolitis, and small bowel transplant. It is associated with significant morbidity and mortality. Interruption of blood supply to a local area causes ischemia which rapidly damages metabolically active tissues. The restoration of blood flow or reperfusion is necessary to maintain cell function and viability, but alone it elicits a cascade of adverse reactions that paradoxically injure tissues. The pathophysiology of ischemia/reperfusion (I/R) injury is complex, involving many biochemical pathways (1–3). Local and systemic inflammatory derangements occur after I/R (4). Damage to the microcirculation triggers a brisk local, then systemic, inflammatory response (5,6). Several mechanisms have been proposed to explain the tissue injury that results from intestinal ischemia. However, little progress has been made in improving the clinical outcome for this devastating disease. The development of novel and effective therapies are imperative in improving patient outcome in gut I/R injury- related conditions.

Studies in gut I/R patients and animals have demonstrated that a key aspect of gut I/R injury is the increased occurrence of apoptotic cell death in the gut (7–9). A large number of studies have shown that excessive apoptosis has pathological consequences on the immune system (10–18). Without proper clearance, apoptotic cells undergo secondary necrosis and have the potential to pose great harm to the host. Milk fat globule-epidermal growth factor-factor 8 (MFG-E8), a secretory protein, is a crucial molecule for apoptotic cell clearance (19–21). Our recent studies have shown that the administration of either MFG-E8-containing exosomes or recombinant murine MFG-E8 (rmMFG-E8), reduces apoptosis and inflammation under various disease conditions (22–24). However, it remains unknown whether MFG-E8 ameliorates bacterial translocation and promotes tissue repair after gut I/R. The purpose of this study was to determine whether MFG-E8 reduces bacterial translocation and promotes tissue repair in a mouse model of gut I/R.

## Materials and methods

### Experimental animals

Adult male C57BL/6J mice, purchased from Taconic (Albany, NY), were used in this study. The mice were housed in a temperature-controlled room on a 12 h light/dark cycle and fed a standard Purina rat chow diet. The mice were fasted for 12 h prior to the procedure. Animal experimentation was carried out in accordance with the Guide for the Care and Use of Laboratory Animals (Institute of Laboratory Animal Resources). This project was approved by the Institutional Animal Care and Use Committee (IACUC) of the Feinstein Institute for Medical Research.

### Experimental model

Ischemia was induced in male C57BL/6J mice (BW, 20–25 g; Taconic) by clamping the superior mesenteric artery (SMA) for 90 min under general anesthesia using isoflurane. At 90 min after SMA, the vascular clamp was released to allow reperfusion. At the beginning of reperfusion mice were resuscitated with a 0.5-ml intraperitoneal (i.p.) injection of saline and were i.p. treated with recombinant murine MFG-E8 (rmMFG-E8; R&D Systems, Minneapolis, MN) at a dose of 0.4 mg/20 g BW in 0.5 ml normal saline or normal saline (Vehicle). The isoflurane was discontinued after i.p. injection of rmMFG-E8 or saline. Control animals underwent the same operative procedure with the exception of the SMA clamping (Sham). Four hours after reperfusion, animals were anesthetized and blood and small intestinal samples (non-necrotic areas; they were selected based on the color of the small intestine segment) were harvested for various measurements.

### Measurement of MFG-E8, Bcl-2, poly (ADP-ribose) polymerase-1 (PARP-1), and vascular endothelial growth factor (VEGF) protein levels

MFG-E8, Bcl-2, cleaved PARP-1 and VEGF protein levels in the small intestine were measured by western blot analysis. The band densities were normalized by β-actin with the use of the Bio-Rad Image System. Briefly, 25 μg of protein from gut samples was fractionated on a Bis-Tris gel and transferred to a 0.22-μm nitrocellulose membrane. Blots were blocked with 5% BSA in Tris-buffered saline containing 0.1% v/v Tween-20. The membranes were then incubated overnight at 4°C with the primary antibodies as obtained from respective vendors: rabbit anti-mouse MFG-E8 polyclonal antibody (1:1,000; R&D Systems), rabbit anti-Bcl-2 antibody (1:500; Santa Cruz Biotechnology, Inc., Santa Cruz, CA), rabbit anti-cleaved PARP antibody (1:300; Cell Signaling Technology, Inc., Danvers, MA), and rabbit anti-VEGF antibody (1:500; Santa Cruz Biotechnology, Inc.). The blots were then incubated with horseradish peroxidase-linked anti-rabbit immunoglobulin G (1:10,000; Cell Signaling Technology, Inc.,) for 1 h at room temperature. A chemiluminescent peroxidase substrate (ECL; Amersham Biosciences, Piscataway, NJ) was applied according to the manufacturer’s instructions, and the membranes were exposed briefly to radiography film.

### TUNEL assay

The presence of apoptotic cells in the small intestine was demonstrated using a green fluorescence-tagged terminal deoxynucleotide transferase dUTP nick-end labeling (TUNEL) staining kit (Roche Diagnostics, Indianapolis, IN) counterstained with propidium iodide and examined under a fluorescence microscope. Apoptotic cells appeared as green fluorescence on a red background staining.

### Histopathology

Samples of the small intestine were fixed in 10% formalin and embedded in paraffin. Tissue blocks were sectioned at a thickness of 5 μm, transferred to glass slides, and stained with hematoxylin and eosin. Morphologic examinations were performed using light microscopy.

### Measurement of myeloperoxidase (MPO) activity

MPO activity in the small intestine was determined using the peroxidase-catalyzed reaction. Briefly, tissues were homogenized in KPO_4_ buffer containing 0.5% hexadecyl-trimethyl-ammonium bromide (60°C for 2 h). After centrifuging, the supernatant was diluted in reaction solution and DOD was measured at 460 nm to calculate MPO activity.

### Bacterial culture

The mesenteric lymph nodes (MLN) and blood samples were collected for bacterial culture. Briefly, the MLN complex was harvested and equal amounts of wet tissues were homogenized and briefly centrifuged to remove gross particulate matters. Serial log dilutions of tissue homogenates or blood samples were applied. Five hundred microliters of each dilution was then plated on chocolate agar plates (Fisher Scientific) and incubated at 37°C for 24 h under aerobic conditions. The colony-forming units (CFU) were counted and the results were expressed as CFU per gram of tissue (MLN) or positive rates (blood).

### Statistical analysis

All data are expressed as means ± SE and compared by the Student’s t-test or one-way ANOVA and the Student Newman-Keuls test. Differences in values were considered significant at P<0.05.

## Results

### Intestinal levels of MFG-E8 decrease after gut I/R

**Figure 1.**
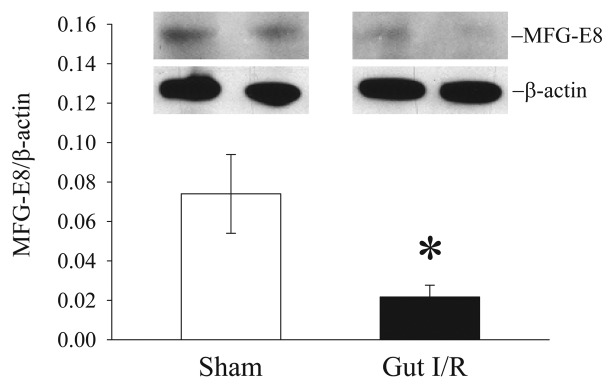
Decrease in intestinal MFG-E8 protein levels after gut I/R. MFG-E8 protein levels were assessed by western blot analysis. A representative gel is presented. Data are expressed as means ± SE, ^*^P<0.05 vs. Sham by Student’s t-test, n=5/group.

To determine whether MFG-E8 levels are altered after I/R injury, we measured its protein levels in the small intestine 4 h post reperfusion after 90 min ischemia. Intestinal levels of MFG-E8 protein decreased by 71% after gut I/R (Fig. 1).

### rmMFG-E8 attenuates intestinal apoptosis after gut I/R

**Figure 2.**
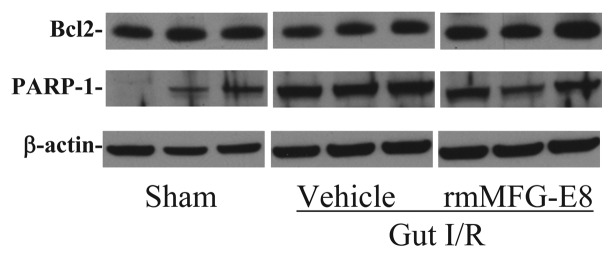
Upregulation of Bcl-2 and downregulation of cleaved PARP-1 by rmMFG-E8 after gut I/R. Small intestinal levels of Bcl-2 and PARP-1 were measured by western blot analysis in sham-operated animals (Sham) and ischemia/reperfusion animals treated with normal saline (Vehicle) or rmMFG-E8 at 4 h after reperfusion. Representative blots were provided.

**Figure 3.**
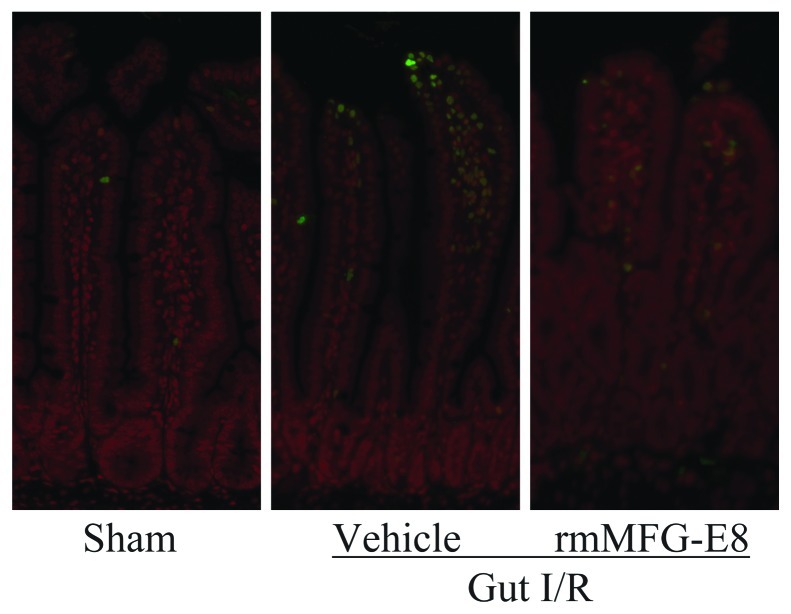
Decreases in apoptosis by rmMFG-E8 after gut I/R. The small intestinal sections were stained with TUNEL (green fluorescence) and counterstained with propidium iodide (red). Photomicrographs of small intestinal sections from sham-operated animals (Sham) and ischemia/reperfusion animals treated with normal saline (Vehicle) or rmMFG-E8 at 4 h after reperfusion. Original magnification, x100.

The intestinal expression of Bcl-2, an anti-apoptosis protein, was markedly decreased after gut I/R (Fig. 2). Treatment with rmMFG-E8 increased intestinal Bcl-2 levels dramatically, which were similar to those in the sham animals. On the other hand, the expression of PARP-1, an indicator of apoptosis, increased dramatically at 4 h after gut I/R (Fig. 2). Administration of rmMFG-E8 reduced intestinal levels of PARP-1 markedly. Consistent with these results, we found an increase in the number of apoptotic cells in the small intestinal tissue by TUNEL staining (Fig. 3). Treatment with rmMFGE8, however, suppressed the number of detectable apoptotic cells in the small intestine after gut I/R injury.

### rmMFG-E8 mitigates intestinal injury after gut I/R

**Figure 4.**
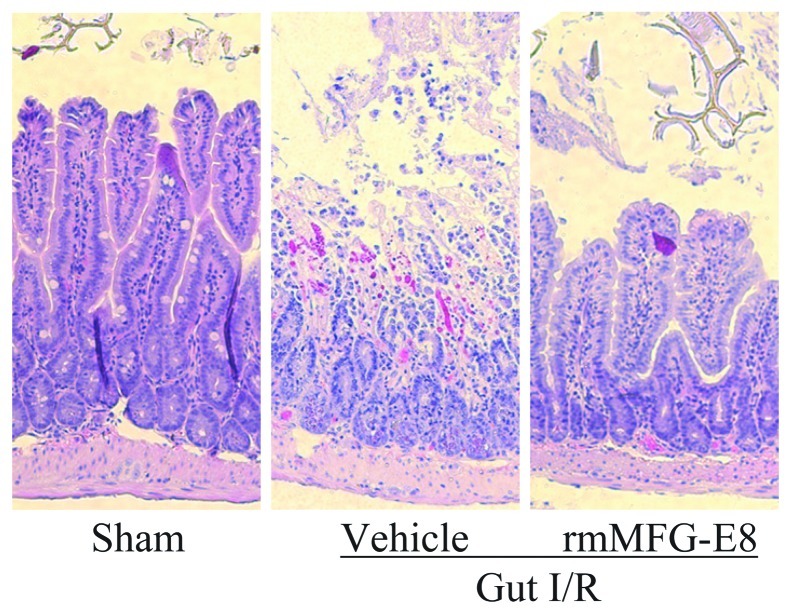
Attenuation of gut injury by rmMFG-E8 after gut I/R. Photo-micrographs of small intestinal sections from sham-operated animals (Sham) and ischemia/reperfusion animals treated with normal saline (Vehicle) or rmMFG-E8 at 4 h after reperfusion. Original magnification, x100.

**Figure 5.**
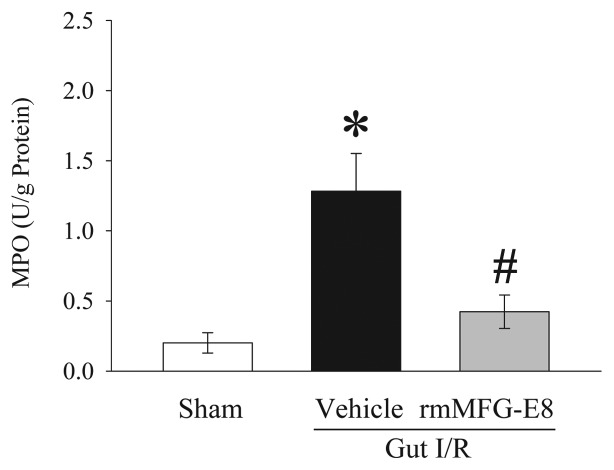
Inhibition of intestinal neutrophil infiltration by rmMFG-E8 after gut I/R. Neutrophil activity was assessed by MPO assay in sham-operated animals (Sham) and ischemia/reperfusion animals treated with normal saline (Vehicle) or rmMFG-E8 at 4 h after reperfusion. Data are expressed as means ± SE, ^*^P<0.05 vs. Sham, ^#^P<0.05 vs. Vehicle by one-way ANOVA and Student Newman-Keuls test, n=6/group.

Mucosal destruction, loss of villi and epithelial cells, hemorrhage, and infiltration of inflammatory cells were observed microscopically in the rat intestine after I/R as compared with sham controls (Fig. 4). Treatment with rmMFG-E8 dramatically improved these microscopic alterations. The level of MPO activity is an indicator of neutrophil infiltration. As demonstrated in Fig. 5, gut I/R induced a more than 5-fold increase in intestinal MPO activities in vehicle-treated rats as compared with sham animals. Treatment with rmMFG-E8 significantly inhibited the increase in intestinal MPO activities by 67% after gut I/R (P<0.05).

### rmMFG-E8 reduces bacterial translocation after gut I/R

**Figure 6.**
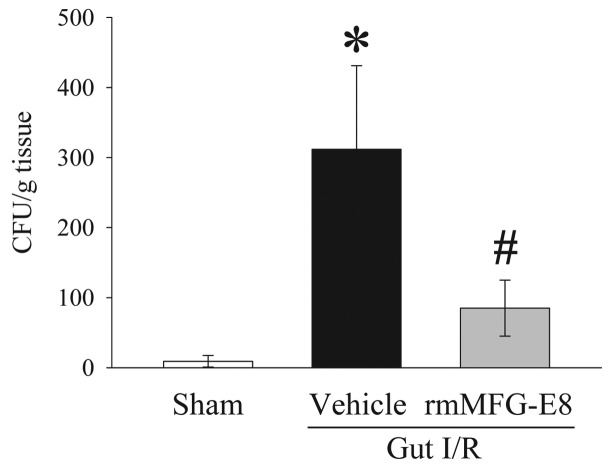
Suppression of bacterial translocation to mesenteric lymph nodes (MLN) by rmMFG-E8 after gut I/R. Bacterial translocation to mesenteric lymph nodes in sham-operated animals (Sham) and ischemia/reperfusion animals treated with normal saline (Vehicle) or rmMFG-E8 at 4 h after reperfusion. Data are presented as means ± SE (n=6), and compared by one-way ANOVA and Student Newman-Keuls test: ^*^P<0.05 vs. Sham group; ^#^P<0.05 vs. Vehicle group.

**Figure 7.**
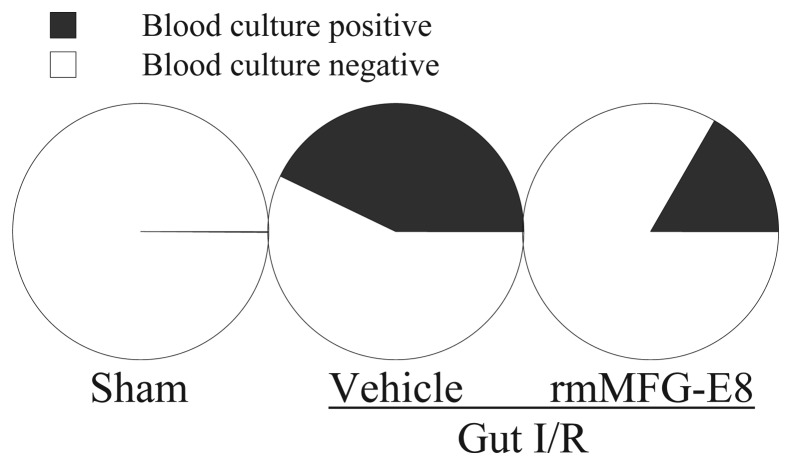
Reduction of bacteremia by rmMFG-E8 after gut I/R. Bacteremia was determined by blood culture in sham-operated animals (Sham, n=6) and ischemia/reperfusion animals treated with normal saline (Vehicle, n=7) or rmMFG-E8 (n=6) at 4 h after reperfusion.

Bacterial translocation to the MLN was minimal in the sham group, but was extensive in the gut I/R vehicle-treated group (P<0.05) (Fig. 6). Treatment with rmMFG-E8 at the time of reperfusion, however, significantly ameliorated the development of bacterial translocation. Moreover, bacteremia was determined by blood culture. As shown in Fig. 7, 3 of 7 vehicle-treated gut I/R animals developed bacteremia at 4 h post reperfusion. However, only 1 of 6 rmMFG-E8-treated gut I/R animals showed a positive blood culture result.

### rmMFG-E8 increases intestinal VEGF expression after gut I/R

**Figure 8.**
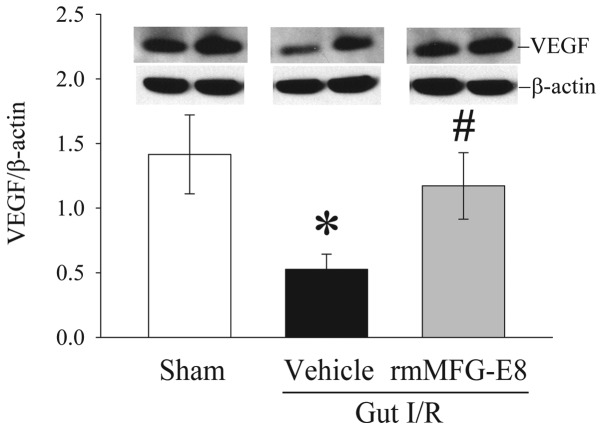
Upregulation of VEGF by rmMFG-E8 after gut I/R. VEGF protein levels in the small intestine were measured by western blot analysis in sham-operated animals (Sham) and ischemia/reperfusion animals treated with normal saline (Vehicle) or rmMFG-E8 at 4 h after reperfusion. Data are presented as means ± SE (n=4), and compared by one-way ANOVA and Student Newman-Keuls test: ^*^P<0.05 vs. Sham group; ^#^P<0.05 vs. Vehicle group.

Intestinal levels of VEGF decreased by 63% at 4 h after gut I/R. Administration of rmMFG-E8 at the time of reperfusion, however, significantly increased VEGF expression in the gut by 123% at 4 h after reperfusion (P<0.05) (Fig. 8).

## Discussion

Gut I/R injury is a serious condition in the intensive care units and among vascular surgical patients. A key aspect of I/R injury is the increased occurrence of apoptotic cell death in the gut (7–9). In the current study, we found that intestinal levels of MFG-E8 are significantly reduced after I/R injury, which correlates with increased apoptosis and impaired barrier function. MFG-E8 is a glycoprotein secreted from the glandular epithelial cells in milk fat globules during lactation (25–27). In milk, MFG-E8 acts as an antiviral protein, inhibiting the symptoms of rotavirus infection (28). Recent studies have shown that MFG-E8 is also produced by macrophages and dendritic cells and has been linked to the opsonization of apoptotic cells (20,21,29–31). It plays a crucial role in the clearance of apoptotic cells (19–21). Binding of MFG-E8 to phosphatidylserine (PS) exposed on the surface of apoptotic cells opsonizes them for a complete engulfment by macrophages via α_v_β_3_- or α_v_β_5_-integrins (32). Without MFG-E8, full engulfment and the removal of apoptotic cells cannot be completed (21). In this regard, gut I/R induces apoptosis in the small intestine, and decreases apoptotic cell clearance through the downregulation of MFG-E8 at the same time. The reduced levels of MFG-E8 in the small intestine after I/R injury may contribute to the increased apoptosis under such a condition.

The current study also shows that administration of rmMFG-E8 decreases apoptosis, mitigates bacterial translocation, inhibits neutrophil infiltration, and promotes tissue repair after gut I/R. The most noteworthy function of MFG-E8 is its ability to promote the clearance of apoptotic cells by forming a tether between phagocytes and apoptotic cells. Excessive apoptosis has various pathological consequences. Recent studies have shown that the lack of clearance of apoptotic cells in the spleen potentially leads to autoimmune diseases (20,21). Accumulated apoptotic cells may undergo secondary necrosis. These cells leak their dangerous contents such as cytokines and enzymes, therefore, exaggerating inflammation and potentiating tissue injury under such conditions. Administration of rmMFG-E8 enhances apoptotic cell clearance, and therefore, a secondary (post-apoptotic) necrosis of apoptotic cells is prevented. Hence, the potential harm from apoptotic cells by leakage of their dangerous contents due to secondary necrosis is abrogated.

Organ injury induced by I/R is not necessarily limited to the ischemic organ. The clinical features of gut ischemia originate from both local and systemic responses. Gut I/R injury i s one of the most common causes of gut barrier disruption (33). Loss of the barrier function of the gastrointestinal tract has been implicated as a potential source of multiple organ failure under such a condition. The gastrointestinal tract not only functions as a site for nutrient absorption but also acts as a barrier between the circulation and noxious substances such as intraluminal organisms entering the circulation (34). Maintenance of normal epithelial structure and function is important in preventing transcellular and paracellular movement of large molecules and bacteria (35). Increased intestinal permeability has been reported to be associated with an increased risk of complications, multiple organ failure, or even mortality in critically ill patients (36–38). Our previous study has shown that the administration of rmMFG-E8 attenuates lung injury after gut I/R (24). In this regard, the restoration of gut barrier function by rmMFG-E8 treatment may also contribute to attenuated lung injury under certain conditions.

MFG-E8 appears to play an important role in the maintenance of intestinal homeostasis and the promotion of mucosal healing. In breast milk fed infants, MFG-E8 is involved in the uptake of milk fat globules in the gut (25–27). It is also an important milk mucin-associated defense component that inhibits enteric pathogen binding and infectivity (39). Previous studies have shown that MFG-E8 regulates the migration of enterocytes and intestinal repair (40) and plays a role in VEGF-dependent neovascularization (41). Various studies have demonstrated that VEGF promotes angiogenesis during acute inflammation and ischemia (42,43). VEGF also plays a role in counteracting the local imbalance of fibrogenesis and fibrolysis, leading to an accumulation of immature subepithelial matrix in collagenous colitis (44). Using intravital microscopy of the rat mesenteric microcirculation to measure leukocyte-endothelium interactions, Scalia *et al* (45) demonstrated that VEGF inhibits leukocyte-epithelial cell adherence and the effects of chronic inflammation. In the current study, we found that MFG-E8 treated animals had higher levels of VEGF in the small intestine after I/R injury. Therefore, increasing VEGF production may be a novel mechanism for MFG-E8-promoted mucosal healing after I/R injury.

In summary, using an established animal model of gut I/R such as a superior mesenteric artery occlusion, we showed that intestinal levels of MFG-E8 are significantly reduced after I/R injury, which correlated with increased apoptosis and impaired barrier function. In addition, administration of rmMFG-E8, decreases apoptosis, mitigates bacterial translocation, inhibits neutrophil infiltration, and promotes tissue repair after gut I/R. Thus, enhancing apoptotic cell clearance by rmMFG-E8 can be a novel concept in the treatment of gut I/R injury.
